# “They’re not doing enough.”: women’s experiences with opioids and naloxone in Toronto

**DOI:** 10.1186/s13011-021-00360-3

**Published:** 2021-03-20

**Authors:** Emilie R. Macleod, Iren Tajbakhsh, Sarah Hamilton-Wright, Nancy Laliberte, Jessica L. Wiese, Flora I. Matheson

**Affiliations:** 1 MAP Centre for Urban Health Solutions, St. Michael’s Hospital, 30 Bond Street, Toronto, ON M5B1W8 Canada; 2 Elizabeth Fry Society Toronto, 215 Wellesley Street East, Toronto, ON M4X 1G1 Canada; 3 School of Population and Public Health, University of British Columbia, 2206 East Mall, Vancouver, BC V6T 1Z3 Canada; 4 Dalla Lana School of Public Health, University of Toronto, 55 College St Room 500, Toronto, ON M5T 3M7 Canada

**Keywords:** Qualitative, Naloxone, Opioids, Women, Overdose, Criminal justice

## Abstract

**Background:**

Amid increasing opioid overdose deaths in Canada since 2010 and a changing naloxone access landscape, there is a need for up-to-date research on Canadian women’s experiences with opioids. Studies on Canadian take-home naloxone programs are promising, but research beyond these programs is limited. Our study is the first to focus on women’s experiences and perspectives on the opioid crisis in Ontario, Canada’s most populous province, since the opioid crisis began in 2010.

**Objective:**

Our objective was to address research knowledge gaps involving Canadian women with criminal justice involvement who use opioids, and identify flaws in current policies, responses, and practices. While the opioid overdose crisis persists, this lack of research inhibits our ability to determine whether overdose prevention efforts, especially involving naloxone, are meeting their needs.

**Methods:**

We conducted semi-structured, qualitative interviews from January to April 2018 with 10 women with experience of opioid use. They were recruited through the study’s community partner in Toronto. Participants provided demographic information, experiences with opioids and naloxone, and their perceptions of the Canadian government’s responses to the opioid crisis. Interviews were transcribed verbatim and inductive thematic analysis was conducted to determine major themes within the data.

**Results:**

Thematic analysis identified seven major concerns despite significant differences in participant life and opioid use experiences. Participants who had used illicit opioids since naloxone became available over-the-counter in 2016 were much more knowledgeable about naloxone than participants who had only used opioids prior to 2016. The portability, dosage form, and effects of naloxone are important considerations for women who use opioids. Social alienation, violence, and isolation affect the wellbeing of women who use opioids. The Canadian government’s response to the opioid crisis was perceived as inadequate. Participants demonstrated differing needs and views on ideal harm reduction approaches, despite facing similar structural issues surrounding stigma, addiction management, and housing.

**Conclusions:**

Participants experienced with naloxone use found it to be useful in preventing fatal overdose, however many of their needs with regards to physical, mental, and social health, housing, harm reduction, and access to opioid treatment remained unmet.

**Supplementary Information:**

The online version contains supplementary material available at 10.1186/s13011-021-00360-3.

## Background

Opioid overdose deaths have increased dramatically across North America over the past decade, stemming from a combination of over-prescribing of addictive opioid painkillers and fentanyl contamination in the illicit opioid supply [1–4]. Canada reported 8.8 opioid overdose deaths per 100,000 in 2016, increasing to 12.3 per 100,000 in 2018 [4]. In total, in Canada, from January 2016 to March 2019, an estimated 12,800 people have died of opioid overdose [4]. The epidemic continues to claim lives despite no official federal declaration of public health emergency [5], with Canadian policymakers resistant to institutionalized, large-scale harm reduction interventions [6]. The lack of opioid research focused on women has only recently begun to receive broader recognition [7], and this lack is especially felt in Canada.

A few recent studies on Canadian women’s opioid use have emerged from Vancouver [8–12], but Vancouver represents a unique risk environment where drug use is concentrated in an area of single-occupancy residences with high poverty [13]. Research from Vancouver does not necessarily reflect the environment elsewhere in Canada, as Canada’s health system is organized by province. There remain substantial gaps in research involving Canadian women who use opioids outside of British Columbia. For instance, the opioid overdose crisis has strongly affected Toronto, Canada’s largest city, with 308 people dying of overdose in 2017 alone [14]. In August of 2017, activists established an unsanctioned but highly needed overdose prevention site in a local park, when no official sites had yet been approved, and Toronto subsequently became the first major city in Eastern Canada to establish an overdose prevention site [15, 16]. Toronto represents an interesting and evolving harm reduction environment where the study of women who use opioids is sorely absent. In Ontario, a thesis compared sex differences in opioid use based on systematic reviews, data from the Genetics of Opioid Addiction (GENOA) study, and qualitative accounts from 492 men and women who use opioids [17]. Results showed female participants were more likely to be unemployed, to report more physical and psychological difficulties than men and to be exposed to opioid use through a prescriber; however this thesis was written prior to Canada’s spike in opioid overdose deaths, and this information may have changed since. One other study compared women’s and men’s opioid-related deaths in 2017, finding overdose deaths ruled as suicides in Ontario made up 9% of men’s, compared to 19% of women’s deaths [18]. Women who died of overdoses were also more likely to have taken benzodiazepines, antihistamines, and antidepressants compared to men [18], which may connect with their mental health burden [17]. Toronto has seen opioid overdose death counts hold steady since 2016 [14]; this contrast of steady deaths with the lack of up-to-date women’s opioid use research in Ontario is of great concern.

Internationally, research into women’s opioid use has also received only limited attention and is focused overwhelmingly on the effects of opioid use on pregnant women, pregnancy outcomes, and neonatal wellbeing [19–39]. While this research is valuable to support the care of pregnant women who use opioids, it excludes women who are not pregnant or cannot get pregnant, and focuses mainly on the physical health of women and the babies they are carrying, not their life experiences and perspectives. International qualitative studies on the stigma against women who use opioids [40, 41], women’s opioid use with intimate partners [42, 43], the intersection of opioid use and sex work [44, 45], women who use opioids in a shelter context [46], and other health outcomes for women who use opioids [47], can at best hint at the experiences of Canadian women who use opioids, but are no substitute for direct research.

Many additional interconnected environmental factors can render women more vulnerable to opioid overdose and death [13], such as criminal justice involvement, opioid criminalization, housing instability, social isolation, and Indigenous status. Mortality is high among men and women leaving prison and overdose is a leading cause of these deaths [48–50]. A recent systematic review and meta-analysis found a pooled prevalence estimate of drug use disorders of 51% among incarcerated women, compared with 1.9% of women in the general US population [51]. One reason suspected for the increase in deaths after release from prison is a reduction in physiologic tolerance after a period of abstinence [52, 53]. Women who have criminal justice involvement and use opioids struggle additionally with the ongoing criminalization of opioids; recent research among people incarcerated in the United States showed harsher criminalization of opioid-related activities further stigmatized the behavior of people who use opioids, without improving their safety [54]. The Ontario government has delayed the opening of additional safe injection sites [55, 56], despite evidence that they can prevent overdose deaths and connect people who use opioids to other services [57]. These barriers stand in major contrast to Portugal, where drug decriminalization in response to a rise in overdose deaths in the 1990s resulted in halved annual opioid overdose deaths, a reduction in opioid use initiation, and a 94% increase in uptake of opioid treatment [58, 59]. A better understanding of how these barriers affect Canadian women who have criminal justice involvement and use opioids can help to inform ways to address the high mortality in this group.

People who use opioids in Canada have also criticized policymakers for their reluctance to address structural causes of the opioid crisis related to poverty and housing [9, 10, 60]. The issue of stable and affordable housing has been raised as a driver of opioid use in the United States [46, 61], and when people who use opioids are also socially isolated or live alone, the risk of overdose spikes [62, 63]. Most overdose deaths in Toronto have occurred in private dwellings, and slightly more than half while the person is alone [14]. A lack of social network, shame, withdrawal symptoms, and financial circumstances can all push people to use opioids alone, even when the dangers of doing so are well-understood [64]. A recent intervention to introduce naloxone training into single-room occupancy hotels in Vancouver helped engage tenants who used opioids and felt isolated, but poor building conditions, stigma from building managers, and criminalization of opioid use acted as barriers to implementation [62]. Another Vancouver housing-based overdose prevention intervention was not accessible to female tenants as they did not feel safe using it [9, 10]. Finally, police discrimination against Indigenous people in Canada [65] further complicates connections between opioid criminalization, criminal justice involvement, and other overdose risk factors. When a community-developed opioid treatment program in a northwestern Ontario First Nations community took the focus off policing, addiction-related medical evacuations were reduced and community participation improved, demonstrating a link between policing of Indigenous people, opioid use, and overdose [66]. There is a high risk of death and a strong need for access to naloxone to prevent untimely deaths among these vulnerable groups. Research with women experiencing some or all of these factors is urgently needed to prevent additional deaths.

Naloxone, sometimes known as Narcan®, is an opioid overdose antidote available in both injectable and nasal forms which, in blocking the brain’s opioid receptors, can reverse potentially fatal overdose effects such as respiratory inhibition. Naloxone availability in Ontario and in Canada more broadly has evolved significantly in recent years, between the creation and expansion of take-home naloxone programs in major cities, and federal adjustments to dispensing requirements allowing access to the medication without a prescription as of June 2016 [67, 68]. However, regional variabilities in naloxone uptake by pharmacies and local populations [69, 70], along with American research indicating naloxone awareness, use, or carrying of a kit is not always commensurate with increased availability [71], point to a need to understand whether or how women in Ontario are learning about, accessing, and using naloxone. This is especially necessary in light of the 2017 Toronto Overdose Action Plan stressing the city’s need for expanded naloxone access [72], as well as international research, mostly involving men, pointing to issues or difficulties people may have surrounding its usage, such as strained peer relationships, precipitated opioid withdrawal, and a lack of overdose aftercare [73–76]. Naloxone is a safe drug, but any unacknowledged gaps in naloxone access, knowledge, and use in Canada may affect overdose outcomes. Given naloxone’s lifesaving importance, research is needed to understand whether or how women who use opioids are able to navigate these barriers in Ontario’s current context, and where different or tailored approaches may be needed.

Women have received almost no focus in Canadian opioid research, especially outside of British Columbia, and even fewer studies explore experiences of opioid use among women with criminal justice involvement and other intersecting socioeconomic vulnerabilities. While earlier studies begin to shed light on issues facing these groups, they fail to achieve a greater depth of understanding of the specific needs, experiences, and ongoing barriers to care facing Canadian women who use opioids, hence the need for qualitative research in this area. The aim of this study is to begin to address these gaps in knowledge among women in Ontario who use opioids and have criminal justice involvement. The study will also simultaneously begin to address a research gap on naloxone knowledge and use among women experiencing criminal justice involvement and who are particularly vulnerable to opioid use and overdose deaths from opioids. In so doing, we hope to inform health professionals, policymakers, social workers, and counselors about the potentially unique or unaddressed challenges facing women with criminal justice involvement who use opioids and naloxone in an urban Canadian environment.

## Methods

### Setting

This study was conducted in Toronto, Ontario, a city strongly affected by the opioid overdose crisis. Interviews were conducted from January to April 2018, providing a snapshot of a unique point in time in Toronto’s initial scale-up of overdose prevention efforts.

### Approach

This study was designed using a community-based participatory research (CBPR) approach to connect with women with criminal justice involvement who had direct experience using opioids [77, 78]. CBPR links academic researchers with local community partners to assess public health disparities, the findings of which can be used to assist in relevant and sustainable community health improvement efforts. CBPR prioritizes the needs of the community being researched by providing a platform to those most familiar with the issue, involving community workers in study design, and presenting results back to the community.

### Eligibility criteria and recruitment

Participants were recruited through a local community organization that supports women with a history of criminal justice involvement. Potential participants were informed of the study during their routine in-person visits with counselors over a 4-month period (January to April 2018), and through an informational pamphlet provided to counselors. Interested clients could volunteer to participate in an in-person interview with a research team member (ERM) if they met the inclusion criteria of identifying as a woman, aged 18 years or older, and with self-reported previous or current opioid use experience.

### Data collection methods

Qualitative face-to-face interviews were conducted with 10 women at the community organization’s primary location. Interviews ranged from 30 to 60 min, depending on the level of detail each participant was comfortable providing. The interviewer had no prior relationship with any participants, and participants were notified of the interviewer’s student status and the study goals during the informed consent process.

Participants provided verbal consent to maintain their anonymity, and interviews were audio-recorded and transcribed verbatim. Counselors were available on-site to support participants if needed. Participants received $20 gift cards and transit fare for their time and reflections. Transcripts and results were not returned to participants for feedback, as personal contact information was not gathered by the researcher, again to maintain anonymity. All participants completed the interview and there were no early withdrawals from the study. This study was approved by the Research Ethics Board at St Michael’s Hospital.

### Interview guide design

The interview guide (see Additional File 1: Qualitative interview guide) contained four sections: socio-demographic characteristics, opioid experiences, naloxone experiences, and perspectives on the opioid crisis. The organization’s staff provided input into the structure and content of the interview guide, and they approved the guide before it was used in the interviews. The interview guide was pilot tested.

### Analysis

Study transcripts were evaluated by thematic analysis [79, 80] using an inductive approach [81]. Participant answers were compared by organizing answers to each question in Excel spreadsheets to see where commonalities in experience emerged. Some answers were further compared based on how recently participants had used an opioid, category of opioids used (licit or illicit), and whether the participant had experienced an opioid overdose; these comparisons were not intended to make any quantitative statements on the data but to better understand specific factors relating to usage, such as participant awareness of naloxone.

Memos were written by the primary analyst (ERM) throughout the analysis process to track emerging insights. Common responses amid individual questions were grouped according to recurring sub-themes during research team meetings involving all co-authors. An inductive approach was used to connect responses across the interviews and incorporate related sub-themes to create the major overarching themes presented herein. Due to small sample size, numbers smaller than 6 are not reported in the results to maintain participant confidentiality. This study was conducted as part of a 4th year undergraduate independent study. The sample size was selected to ensure that the project could be completed within the course requirements. It is possible that saturation was not achieved. The authors completed a check list of criteria for reporting qualitative research (See Additional File 2: COnsolidated criteria for REporting Qualitative research checklist).

## Results

### Participant characteristics

Participants ranged in age from 27 to 60, with an average age of 42. A majority were White, with a minority of participants identifying as Indigenous. The study featured both cisgender and transgender participants. All participants were low-income and several had less than high school education. Most participants had fixed addresses, but some did not. Each participant had been living with at least one mental or physical illness and most participants had multiple chronic conditions, including post-traumatic stress disorder and/or chronic pain. Nearly all participants were taking at least one non-opioid medication, with some concurrently taking five or more medications.

The most commonly used opioid reported by participants was heroin, followed by Percocet, Oxycontin, and morphine. Participants also mentioned other opioids including Tylenol 3, codeine, fentanyl, methadone, hydromorphone, or unspecified pills. The majority of participants used more than one type of opioid with many having used three or more in their lifetime. Participants reported that cocaine and crack were the most common substances combined with opioids, followed by alcohol. The most frequently cited reasons for combining opioids with other substances was to experience new sensations, to come down from a high, manage pain, or numb negative emotions. Nearly all participants knew at least one person who had experienced an opioid overdose.

### Naloxone knowledge and experience in relation to opioid use patterns and pathways

Each participant described a unique initiation into opioid use, with many experiencing more than one extended phase of opioid use over the course of their lives. Some participants experienced an initial period of prescription opioid use for illness or injury, followed by a phase of injecting heroin. For instance, one participant was prescribed Percocet in her mid-20s, ceased use, then began injecting heroin in her late 20s with a partner. Other participants used heroin and opioids recreationally and later were prescribed these drugs to address health conditions; one participant had smoked heroin while younger, then was prescribed opioids in middle age for degenerative disc disease. Participants who experienced only one phase of opioid use were initiated through prescriptions, pressure from romantic partners, or using with friends.

Participant awareness of and possession of naloxone appeared to relate to how recently they had used an opioid and the type used. Many participants were using opioids at the time of study or during the preceding year, and most participants with recent opioid use were aware of naloxone. Only one participant who had recently used opioids reported not ever having a naloxone kit; this participant had taken a low-dose prescription opioid and did not display a pattern of recent illicit use. All other participants engaged in current or recent opioid use, and aware of naloxone or in possession of it, had been using opioids illicitly, primarily heroin. Most participants engaged in illicit opioid in more recent years had received a naloxone dose, some from take-home naloxone kits, others from emergency medical personnel for both suspected and actual opioid overdose. Many were knowledgeable of both injectable and nasal forms of naloxone and had used naloxone on another person at least once.

Conversely, participants not engaged in recent illicit opioid use at the time of interview, who in some cases had not used opioids in several years, either did not know about naloxone prior to study recruitment, or had only heard of it in passing, such as on the news, without pursuing additional training or knowledge. No one in this group had administered naloxone to another person, and when asked, many were not interested in acquiring a kit. One participant who had not used opioids in more than a year at the time of interview had previously been prescribed Narcan® but had not used it.

Participants engaged in illicit opioid use since 2016 described obtaining multiple naloxone kits and information from a variety of sources: primarily friends, partners, community or public health services, methadone clinics, and pharmacies. Some participants turned to the news or online research for additional information on naloxone. No participants reported initially learning about naloxone at a pharmacy. The decision to seek out naloxone training was often a product of the initial awareness of naloxone they had gained from peers:

“I lived in a house where a lot of people were using IV drugs. A lot of people came there to use IV drugs, so- and nobody was being careful. So, I took it upon myself that I always had- I was the one to call [public health service] to get clean kits, fits, everything.” (Participant 6)

Despite a lack of knowledge of naloxone, a few participants with earlier-life heroin use who were later prescribed opioids for medical reasons consciously limited their prescription use. These participants tapered off their prescriptions or avoided taking them unless absolutely necessary, citing a fear of becoming addicted, the desire to pursue Indigenous spiritual and mindfulness-based pain management practices, and side effects as reasons for their self-imposed restrictions. These participants had not experienced accidental opioid overdoses (though one had intentionally overdosed in the past, and one had intentionally overdosed on a non-opioid) and did not express interest in acquiring naloxone kits.

Participants who had described recent heroin use, regardless of whether they had been prescribed opioids in the past, had experienced accidental overdoses prior to the interview. Although naloxone did appear to be reaching these participants, who were in need of it, the ever-present danger of overdose loomed:

“I went to the pharmacy to fill my medication and the day before I had- I ran into a guy overdosing on the street, and a week before that a girl overdosed on the TTC and I- at which point I didn’t have any of the- naloxone or anything and I just realized that, whether I want to see an epidemic or not, it is happening in my cities … so … I just picked one up.” (Participant 5)

Participants engaged in more recent (post-2016) illicit opioid use were the most experienced with naloxone, and tended to seek out naloxone knowledge as a means of mitigating their overdose risks, with some relying on their informal social networks to access and learn about naloxone. Participants who had used illicit opioids in the past and then were later prescribed opioids for medical reasons were not very aware of nor interested in naloxone kits, even if they had overdosed in the past, but consciously limited their use of medication in other ways. These differences in naloxone usage and knowledge across specific opioid initiation and use pathways were consistently apparent in the data.

### Naloxone’s dosage form, effects, and ease of use

Injectable naloxone kits were available in Ontario prior to Narcan®, the nasal spray form. These kits, the size of a large wallet, contain two ampoules of naloxone, two syringes, gloves, alcohol swabs, and instructions. Nearly all participants were supportive of naloxone use, but when asked what they found positive or negative about the kits, one participant said their bulkiness is an impediment to women carrying them:

“ … if they don’t fit in our purse … chances are it’s gonna be sitting at home, somewhere in a safe spot but not where you need to have it … like a regular epi pen like keep it in your tampon container in your makeup bag or in your purse and, it’d be a lot more effective.” (Participant 5).

Multiple participants drew comparisons between naloxone and epi-pens as a fast-acting, portable emergency medicine. When asked, participants experienced with naloxone use were inclined to prefer the nasal spray form over injectable forms due to ease of set-up, as well as fear of needles or discomfort surrounding needle size.

One participant described severe naloxone-induced withdrawal after an overdose, after which she used additional opioids to manage her symptoms. These withdrawal effects greatly distressed her, and she expressed a need for greater support for people immediately post-overdose:

“ … it brings you out of your overdose, but at the same time, you get extremely sick, you know? Like it takes out every, every opiate in your body and you’re basically in withdrawal and then like what if- what if that was the last of your money or your dope and now you’re gonna have to go find something else and- and you’re so sick that you don’t even want to do anything, you know? And nobody helps. So it’s- that’s the only part I fuckin’ hate about those kits.” (Participant 3)

Another study participant, who supported the above participant through her overdose, independently shared her belief that health officials and authorities lack awareness of naloxone’s effects and how to best manage them:

“I believe doctors, police officers, jail people, they are not knowledgeable enough about how this drug [naloxone] affects people and what happens to them when they go through withdrawal.” (Participant 2)

Some participants identified the dosage form of naloxone, as well as its effects, as points of anxiety, discomfort, or even distress. The portability of kits was also identified as a concern by one participant. Despite this, most participants expressed a positive view of naloxone.

### Gendered violence, alienation, stigma, and the role of social support

When asked if they had experienced gender-based discrimination when accessing health, naloxone, or harm reduction services, most participants answered no; however, one participant highlighted difficulties resulting from a lack of services focused on women:

“ … when I got in trouble, and got arrested, the only place in the world that could help me was here … And, it was like there’s not enough places out there for women. And, prior to me, I was screaming out for help.” (Participant 4)

Even though most participants answered that they did not experience discrimination in harm reduction contexts, they did report conflicted self-perceptions over their opioid use, strained family relationships, social isolation, and instances of violence against women who use opioids. Participant responses painted a sense of collective alienation with regard to their roles as women and their place in society:

“… I guess I feel that women are supposed to have their shit more together or we’re expected to be of more moral and more, higher standards than- do you know what I mean? … Yeah, the motherly figure, right? Like what the hell’s a mom doing shooting up or asking for naloxone, you know?” (Participant 1)

This experience of stigma was reflected in other participants’ stories of fractured relationships in association with their opioid use:

“I had a high paying job. I drove a really nice car. I owned a home. I was a single parent to my daughters. And one by one, I lost, absolutely everything.” (Participant 2)

“My daughter, my wife. I’d pick on them, the cocaine would pick on them, the Oxys- or the opiates would pick on them. And, when I’d be around them I’d be miserable.” (Participant 4)

Another participant, who identified as transgender, felt her estrangement from her family was due to a combination of disclosing her drug use, and her transition to trans status at an older age:

“Yeah it was- it caused a lot of problems and distrust and just owning up- you know what I mean? ... afterwards you know my family tried to disown me and, doesn’t want anything to do with me now pretty much ... and especially now I think that I’ve turned trans because I lived [age] as a heterosexual male and I just started transitioning like 2 years ago so.” (Participant 7)

However, these negative self-images and experiences were not uniform across the study participants; one participant described her catharsis when her mother was ultimately supportive of her recovery:

“I don’t know how it came out. We were talking, then it went from talking, to yelling, to me screaming and crying “I’m a fuckin’ IV drug user!” and my mom’s like “I’m not understanding!” and, I’m like “I SHOOT NEEDLES IN MY ARM!!” and she was like- then you know, then she broke down with me and then she became my biggest supporter.” (Participant 1).

Participants felt their social lives were deeply affected by their opioid usage. Some participants avoided disclosing their opioid use to others but felt as though they would inevitably be found out. Others described how social pressures from opioid-using friends or intimate partners either initiated or perpetuated their opioid use. Multiple participants described a shift in opioid use over time; from using opioids with others, such as at parties, towards using opioids in smaller groups or alone. Some participants explained this shift as a result of a lack of trust in people who use drugs, a desire to keep drugs for themselves, or a sense they would be judged for how much they were using, sometimes to a point where they avoided connections with other people who use opioids.

Participants described two instances where perceived stigma against women who use opioids was so severe as to result in violence. One involved an incident on public transport:

“ … there was this girl, front of the bus, clearly she was not drunk, she was OD’ing you know what I mean? Like, all the signs were there. The bus driver kicked her. Then he kicked her again. Then he said “Hey- hey you, you damn drunk! You’ve got to get up off my bus.” I said “She’s not drunk. You need to phone 911.” He’s like “How do you know?” I was like “Cause I’m a junkie you asshole”.” (Participant 1).

Another participant recounted a violent altercation with emergency and hospital personnel after her partner had suffered a non-opioid overdose while opioids were still in her system. Medical staff administered naloxone, and her partner went into opioid withdrawal:

“As soon as they [medical staff] Narcan’d her, w-well- whatever you call it- all the drugs came out of her system so as soon as she went through- through- with this- what do you call that, with-withdrawal. Yeah, so she wanted to leave, right away, to go and get high. And they wouldn’t let her go, and so she started like- flipping out and. They [personnel unclear] tied her down, they beat her … she had bruises on her face, everything.” (Participant 3).

Participants and those around them experienced social alienation, and often rejection from past support networks. Participants also described dangerous situations they or other women had experienced while using opioids. Despite these conditions, participants did not feel discriminated against while accessing harm reduction services, and some participants expressed that they benefited greatly from the continued support of their families.

### Government response to the crisis

When asked about the municipal, provincial, and federal governments’ responses to the opioid crisis, one participant stated it was “kind of disgusting.” (Participant 2). Most participants felt that the response at every level of government was too slow, and reflected negative public sentiment surrounding opioid use. When asked whether the overdose crisis should be declared a provincial public health emergency, nearly all participants thought so, with multiple participants additionally stating they wanted a nationwide, not just provincial, emergency declaration. They felt that a lack of desire by politicians to help, rather than an inability to help, was making the crisis worse:

“We live in Canada. We have access to free healthcare. And yet there’s people that are suffering. Even for rehab, you know? W-we have to wait and go on waiting lists, and we have to cut through all this red tape. It’s bullshit. Absolute bullshit. Somebody wants help, they want help that day.” (Participant 2).

“If they were so concerned, why didn’t they do it a while ago? Right? Why did people have to die? And a public outcry, for things to change around?” (Participant 8).

“They’re not down you know working with people, face-to-face, interacting and seeing how many people are being affected … they’re very detached.” (Participant 10).

Participants were nervous that not enough was being done about fentanyl in the drug supply. They felt that a lack of adequate opioid addiction treatment services was resulting in unanswered pleas for help and even deaths of people they knew:

“They’re not doing enough at all, they’re not doing enough. My- I had a girlfriend … her nephew passed away waiting for a bed to get in treatment.” (Participant 1)

One participant, who tried methadone treatment, returned to heroin as she felt the dose was too low. She experienced severe withdrawal symptoms and felt she had no support:

“They start you off at a really low dose, and because I was using such high doses of heroin that I would still feel sick like I would still be like vomiting, my legs would still ache.” (Participant 3)

Participants described how they felt economic issues, when unaddressed by different levels of government, could perpetuate opioid use and keep people at risk of overdose. One topic that came up spontaneously from multiple participants were concerns over housing availability and pricing in the Greater Toronto Area. Nearly all participants were in precarious housing situations with limited incomes, giving them a very personal insight on this issue:

“I think the number one problem with the opioid crisis is that, a lot of it affects homeless people and … they can get better rehab but we put them back on the streets with the same problems in the same situations, they’re gonna go to what’s known to them and that’s the drugs.” (Participant 5)

“These people don’t necessarily wanna live on the street, but there’s nowhere else to go, for them. With disability you only get so much money, and you can’t afford a house or an apartment, because the prices in Toronto’re skyrocketing … you can’t get a- a basement apartment under 800. One-bedroom. A bachelor’s! Like it’s ridiculous. How do you live? Where do you live? … I was shocked to hear yesterday, since the beginning of December, 70 people died on the streets so far … I just think the lower class is getting’ rubbed into the ground now.” (Participant 4)

Participant responses were not entirely negative; some praised the Moss Park initiative, an overdose prevention site established by activists and peers, in the months before officially sanctioned sites began opening in Toronto. Participants also expressed gratitude for government approval of safe injection sites, which began to open while interviews were taking place:

“I think it’s amazing that they have safe sites. Where I come from that’s not an option. Like I said, drug use is very behind closed doors … I think Toronto’s taking a good approach to having safe sites. Not only do people use there, they feel safe there.” (Participant 1)

When asked about the government’s response to the opioid crisis, participants’ responses prioritized ongoing, unaddressed structural causes of the epidemic (e.g., the housing crisis, perceived stigma, and a dearth of opioid management services) and the lack of motivation by government officials to address the issue in a reasonable time frame or listen to those who are suffering most.

### Preferred emergency personnel for overdose response

Participants were asked how they felt about fire services, police services, and paramedics carrying naloxone. Most felt all responders should carry naloxone, and some even went so far as to say they should be readily available in other public spaces as well.

“Everyone! Everyone should be carrying them, you know what I mean?! They should be in restaurants” (Participant 1)

“All of them should have it. One hundred. I think that a lot of cops on bikes should have them too ... in the summer in Toronto they’re the first one on responses in the streets ...” (Participant 5)

Other participants were unsure about police officers carrying naloxone, believing what they consider the punishment-oriented ethos of law enforcement to be at odds with helping people whose drug use is criminalized.

“Obviously the other ones have like some form of training in health care or, you know- emergency and (sigh). They’re more like concerned about, you know” “Are you okay?” you know are you- like, let’s save the person whereas law enforcement’s like, “Let’s put you in jail!” (Participant 10)

One Indigenous participant reported repeated negative experiences with police that made her suspicious of their ability to deliver appropriate help to people suffering from overdose, and, additionally, believed paramedics overuse naloxone:

“Personally I don’t give- don’t think cops give a fuck...I’ve dealt with them before, they don’t give a fuck. I’ve been dealing with them since I was 12 …. I just think they would let somebody die whereas, I dunno the ambulance... my partner had something happen to her and it had nothing to do with anything to do with drugs or anything and they Narcan’d her...They are not properly trained...I think they overuse it.” (Participant 3)

While some participants were accepting of the possibility of having naloxone administered by police, others were opposed, especially if they felt their overdose would be punished or treated with indifference.

### Views on opioid criminalization and legalization

Participants were asked their views on the legalization of illicit opioids, and expressed a wide range of opinions, but each framed their responses in terms of which option they felt would best protect others from overdose. For some, this meant supporting current restrictions on highly potent opioids like heroin, even if they had used them themselves. Some viewed the prospect of legalizing all opioids to be a safety measure that would reduce stigma and ensure patients know precisely which drugs they are using:

“I think drugs should be legal all the way around. I think, or- have it regulated. Like there’s a European country where people go shoot up pharmaceutical grade heroin and they’ve done tests that, you know the dr- the crime has gone down in that area and I think they should do the same thing here because we’re in an epidemic right now, it’s bad.” (Participant 6)

“I think the system does criminalize it and it-it’s not helping, it’s, like, like that whole punishment, like aspect of things makes you want to do it more, makes you feel worse about yourself, and makes you feel like “Well there is no hope so why would I stop anyway?” … There should be more help when it comes to people who have, addiction I mean like at the end of the day, whether it’s a street drug or a pharmaceutical. Most of- all- everyone in our population use some form of something” (Participant 10)

Other participants were torn on the best approach to legalization; they acknowledged concerns related to opioid misuse, while also stressing their importance in pain management:

“I’m torn because I really believe in the decriminalization and legalization of pot. I don’t think opioids should be legalized for anybody. However, I feel like it’s really being a blurred line … I feel like sometimes it’s harder for people who actually need that to help with their pain.” (Participant 5)

“It- it shouldn’t be given out like candy. It should be labeled to cancer patients and only cancer patients, people that are dying ... people that are in massive massive pain.” (Participant 4)

Each participant framed their views on opioid legalization in terms of what they felt would be the safest option for people who use opioids, but these priorities were expressed in many different ways, depending on the individual. Overall, very few participants outright opposed the legalization of illicit opioids, but not all were in support, either.

### Unique needs, preferences, and coping methods for opioid use

Each participant had a unique approach to processing, managing, or in some cases ceasing their opioid use. A participant, who identified as Indigenous and two-spirit, felt her opioid use had been in conflict with her beliefs, and eventually ceased taking her prescription:

“… I’m really spiritual, I follow my ceremonies and stuff. So it- I don’t feel- I would be a hypocrite [if I continued to take opioids], you know?” (Participant 8)

One participant expressed a positive view of her opioid use in the face of multiple chronic illnesses, explaining how opioids helped her to live a more normal life:

“Oh! I could run, I could jump, I could do what I wanted to do!” (Participant 4)

Some participants felt completely overwhelmed by the impacts their opioid usage had had on their lives:

“It sucks it’s like a full time job it’s like you always have to have your next dose ready or this or that and you have to deal with being sick and. It’s consuming.” (Participant 5)

An older participant acknowledged the negative impact her opioid use had on her life, but explained how she had come to accept the way her experiences had shaped her life:

“… it’s like a waste of my life kind of thing you know but without that experience I wouldn’t be the person I am now. You know what I mean?” (Participant 7)

Participants did not represent a monolith, and each had unique experiences and needs when it came to living with their opioid usage.

## Discussion

This study is the first in Canada outside of British Columbia, and one of a limited number internationally, to specifically examine the experiences and policy perspectives of women who use opioids in the context of the current opioid overdose epidemic. This study also addresses a gap in knowledge within Canadian naloxone research. The research, conducted with people who use opioids directly, is in line with the priorities laid out in the Toronto Overdose Action Plan [72] and other Canadian opioid research indicating the perspectives of people who use opioids requires additional attention [6, 60], and wider calls for additional opioid research involving women [7]. In examining both individual circumstances, experiences, and viewpoints, we garnered a greater understanding of the risk environments faced by women who use opioids, and where current approaches may be insufficient [13]. Such research also has implications for governments and organizations considering the implementation of naloxone kit programs and other harm reduction services, to ensure these services meet the needs of women.

Nearly all participants found the Canadian federal and provincial governments’ responses inadequate in addressing structural influences on present-day opioid use, such as lack of affordable housing, widespread social stigma, and a lack of other opioid services. This inaction has been repeatedly criticized in the literature [6, 60] and our findings reiterate perceptions of government neglect and ongoing suffering. Previous limited research on women’s opioid use in Ontario indicates a high mental health burden [17] and a higher instance of death from intentional overdose [18], in addition to previous research indicating high mortality from overdose among people with criminal justice involvement [48–51]; paired with our findings, we can begin to understand what is driving these vulnerabilities, especially among participants who engaged in opioid use at the time of study. A lack of rapid or broadly-focused interventions left participants in this study feeling ignored and undervalued. The issue of stable housing raised in opioid studies in the US and British Columbia [9, 10, 46, 61] was reflected in our study population. Participants felt the local risk environment in Toronto [13], involving rising rents and homelessness, and low incomes or disability payments, would hamper overdose prevention or opioid treatment efforts. As well, many of our participants struggled with social alienation, and described a shift over time to using opioids alone, which is a significant known risk factor for opioid overdose [62, 63], particularly in Toronto, where most overdose deaths have occurred when people were by themselves in their own homes [14]. Participant reasons for using alone developed in complex ways and mirrored findings of other research on solo opioid use, where a lack of trust in peers, or shame over drug use or the amount of drug used, drove them to avoid using opioids with others [64]. Participants praised naloxone and the existence of overdose prevention sites, including the unsanctioned Moss Park overdose prevention site, as effective harm reduction interventions. In research published since this study was conducted, support for this unsanctioned site has only been reified by people who use drugs in Toronto [15]. However, both the life circumstances and views of our participants indicate, alongside relevant literature, the need for a dramatically scaled-up government response at the provincial and federal level. Concerns around safety were also reflected in descriptions of stigma and violence witnessed by some participants on public transit or in health care settings, indicating a potential need for greater supports for workers in public service environments that are more likely to encounter people experiencing an opioid overdose in a public setting. This could include providing workers with additional tools and education on overdose response and non-violent intervention methods, as well as implementing prevention efforts that lower the likelihood of overdose in the first place.

While multiple provinces have established take-home naloxone kit programs through pharmacies and community health services, with positive results on a program level, high numbers of opioid overdose deaths have persisted in Canada [67, 68]. Variabilities in naloxone access and knowledge have the potential to impact its effectiveness [69, 70]. Only 56% of participants in a recent large survey of formerly incarcerated men and women in addiction treatment in Michigan correctly identified naloxone as an opioid overdose antidote [71]. These variabilities by region, demographics, and through the naloxone use cascade, all highlight a need for further research into specific groups who use opioids, to determine how they acquire naloxone knowledge or perceive naloxone use.

In the mid-2010s opioid overdose deaths and fentanyl poisonings increased significantly and naloxone became available over-the-counter [82]. The differences we observed in naloxone experiences for women who used illicit drugs more recently could have been generated by either increased naloxone access or from the increase in overdoses and overdose risk (e.g., uptake in naloxone education and acquisition among participants). While we cannot say definitively that the scheduling change allowing for over-the-counter naloxone access was the impetus for increased awareness and/or use of naloxone this is an important area of inquiry for future research. Naloxone research in the United States with majority-male samples, for instance, has shown current opioid use, witnessing an overdose, and recent non-fatal experience of overdose were associated with greater naloxone knowledge and possession of a kit [83, 84]. The opportunity for peer-led support in this study population needs to be highlighted, as women often learned about naloxone through informal social networks, instead of through health care providers. Though naloxone use was widely supported by participants, they preferred smaller, portable, needle-free forms such as Narcan®, a naloxone nasal spray that became available in Ontario after the study was conducted. The findings also revealed what women felt was an unmet need for appropriate naloxone-induced withdrawal aftercare and reduction in wait times for opioid treatment services. Only a few recent studies with majority-male samples have examined naloxone-induced opioid withdrawal, but the findings are also consistent with those of our study [73–76]. Participants in these studies often felt unable to provide appropriate care for someone experiencing acute opioid withdrawal symptoms. Naloxone’s lifesaving qualities should not overshadow issues around precipitated opioid withdrawal, both in public health interventions, policymaking, and in its use by medical or emergency personnel. One of our participants described using additional opioids after naloxone-induced withdrawal made her sick and distressed, putting herself at risk for a repeat overdose, but also demonstrating the limits of Ontario’s current approach to naloxone. To our knowledge, Canadian interventions involving post-overdose support for acute withdrawal have yet to be studied. While some of our participants began carrying naloxone out of a sense of responsibility to their community, others, usually those who had not engaged in recent opioid use, were much less familiar with naloxone and not interested in carrying a kit. A qualitative study involving sex workers in Vancouver stressed the emotional toll of frequently being expected to administer naloxone to clients who use drugs [12], and along with our results, we can see how widespread naloxone possession as a well-intentioned public health goal, in the absence of broader overdose prevention or aftercare interventions, shifts an undue burden of care onto individuals who may not always be ready or able to provide assistance. Naloxone does not address the steps leading up to an overdose, and our study revealed difficulties women can have using this method of last resort despite their positive views of the antidote and its ease of access.

Indigenous-identifying participants in our study were all opposed to police services responding to an overdose and administering naloxone, which is consistent with Indigenous experiences of police discrimination in Canada [65] and with the positive results of a community-developed opioid treatment intervention study in a Northwestern Ontario Indigenous community that avoided a police-based approach [66]. Non-Indigenous participants also expressed opposition to police administering naloxone. This skepticism is also apparent in the findings of the Toronto Overdose Action Plan, where 89% of survey participants felt it would be of large or very large benefit for police not to attend overdose calls, and citing Vancouver as an example of a city that has implemented a policy in this vein [72]. Harsher punishments for people who use opioids in the form of stricter drug-induced homicide laws and mass incarceration, in the United States, have worsened the health and wellbeing of incarcerated people who use drugs [54], and this view was mirrored in participants who opposed police as emergency overdose responders. Fear of punishment by law enforcement can cause people who use drugs to avoid calling 911 during a medical emergency such as an overdose [54, 72]; these fears largely relate to the ongoing criminalization of drugs such as illicit opioids. Opioid criminalization is also reflected in the reluctance by the Ontario government to open additional safe consumption sites [55, 56], despite other research indicating such sites lower overdose deaths [57]. Our participants expressed a wide variety of views on the question of opioid legalization, with some in full support of decriminalization as seen in other countries, such as Portugal [58, 59]. Other participants were completely opposed to the legalization of high-potency opioids such as heroin even if they currently or previously used it themselves, as they felt it would enable or endanger people who use opioids further to have easier access. Multiple participants were unsure which stance to take. All participants framed their preferences, in terms of what would provide the safest environment for people who use opioids, as opposed to what they personally wanted, showing concern for their community. Some spoke openly in support of safe consumption sites, even though they were not asked specifically about them because none had yet been approved and opened in Toronto at the time of the study. Based on the participants' responses, a similar community-based approach to the accessible opioid substitution treatment described in Kanate et al. [66] may benefit Indigenous women who use opioids in Toronto.

Delivery of opioid-related services to women will need to consider the unique needs of women, especially those who experience criminal justice involvement [85]. In a paper about women in correctional settings the authors described five guiding principles that are gender-responsive and designed to support women with criminal justice involvement [85]. These include a recognition that gender differences affect pathways to incarceration; that safety, respect and dignity are critical components of policy, programs, and services designed to support and enhance connections to children, significant others and the community; and that services should be holistic, culturally relevant and enhance women’s ability to be financially self-reliant. Racially diverse and gender and sexual minority women require special consideration given their overrepresentation in prisons [86]. Wright et al. [43, 87] echo the need for services to be appropriately responsive to the specific needs of women (gender-responsive framework). This starts with an awareness of the distinctive experiences of women, including pathways to criminal activity and the ways their lives are shaped by relationships.

Our study was designed with the aim of addressing the known gap in up-to-date qualitative research on women who use opioids and naloxone, particularly in Canada. However gaps do persist in research on Canadian women who are not White, rural women, women in Canadian cities outside of Toronto and Vancouver, and incarcerated women who use opioids.

Participants overall showed great awareness of the impacts of the opioid overdose epidemic on their communities and lives and coped with their opioid use in unique ways. While participants faced many common structural issues such as economic and housing precarity, opioid-specific issues such as social stigma, and overdose risks, the decision to continue or stop taking opioids also varied based on their beliefs, their access to addiction management services, and their personal circumstances. In light of the legalization of cannabis under Canadian federal law, which has implications for how Canadians manage pain [88], it is important to consider complex individual factors and comfort levels when discussing pain management practices, overdose prevention methods, and opioid use treatment or management with women who use opioids.

### Limitations

This study has both strengths and limitations. While the sample size was small, this was a pilot study set in an urban environment and the first to explore women’s experiences of opioid use in the context of widespread naloxone use during Canada’s opioid overdose epidemic. The women in the study were mostly older and White, and as such, the experiences of people of younger and of ethnically diverse populations are missing from this study, however a portion of the sample was Indigenous and their specific experiences are captured. Regardless, the themes that emerged from this study may not reflect all women who experience criminal justice involvement and opioid use. The sample was entirely low-income, and so did not account for the opioid- or naloxone-related experiences of middle- or high-income people, however the researchers and participating community organization were particularly interested in understanding opioid and naloxone experiences among this population. These missing perspectives are still needed in research so long as there is an opioid overdose crisis affecting their health outcomes and personal wellbeing.

The study was conducted from January to April of 2018, and may not reflect changes or policy developments that have occurred since. Cannabis legalization, the introduction of Narcan® nasal spray, and the expansion of safe injection sites are all changes that have occurred in Toronto and now affect women who use opioids there. Overdose prevention sites, appropriate emergency response measures, and opioid criminalization remain contentious political issues, and opioid overdose deaths remain a regular occurrence. Despite its limitations, the snapshot provided by this study continues to be relevant within Canada’s current public health context.

## Conclusion

In conclusion, difficulties concerning opioid-related stigma from families and strangers, naloxone-related discomforts, economic barriers to health and housing stability, and complex individual life experiences, all affect the health and safety of women who use opioids. Future, Canadian opioid policy must account for women’s needs around naloxone and beyond in order to prevent additional overdose deaths. This includes adequately informing people who use opioids on all forms of naloxone available to them, expanding access to timely mental health, opioid- and overdose-related care, assisting with the formation of new social support networks among women who use opioids, providing housing to people in economic precarity with complex health needs, keeping safe injection sites open and opening additional sites, and educating not just the public but policymakers and health professionals on opioid-related stigma that may stereotype women who use opioids and deny them dedicated, individually-tailored support. Further, research on women in Canada who use opioids and naloxone, especially ethnically diverse women, Indigenous women, and women in rural areas, is necessary. Current research, including this study, supports the rapid implementation of these recommendations.

## Supplementary Information

**Additional file 1:** Qualitative interview guide.

**Additional file 2:** COnsolidated criteria for REporting Qualitative research checklist.

## Data Availability

All data generated or analysed during this study are included in this published article.

## Abbreviations

GENOA: Genetics of Opioid Addiction study
CBPR: Community-based Participatory Research
