# Supplementary material for: “They’re not doing enough.”: women’s experiences with opioids and naloxone in Toronto
Source: Subst Abuse Treat Prev Policy. 2021 Mar 20;16:26. doi: 10.1186/s13011-021-00360-3 (PMC7980746; doi:10.1186/s13011-021-00360-3)
Supplement: Supplementary file 1 — Additional file 1: Qualitative interview guide. [file 13011_2021_360_MOESM1_ESM.docx]

**Supplemental File 1: Qualitative Interview Guide**

**Women’s Experiences accessing Naloxone in Toronto (WENT) Study Interview Protocol**

Interviewer’s Name:

Participant ID:

Date:

Interview Location:

_________________________________________________________________________

Introduction

You have been invited here today to share experiences you have had with opioid use, as well as with access and use of naloxone services in Toronto. Before we begin, I am going to review the Consent Form and Letter of Information with you. If you have any questions or concerns, feel free to ask at any point.

**[Letter of Information and Consent Form will be reviewed with the participant in order to obtain oral consent. Participants will also have an opportunity to ask any questions or express any comments they may have.]**

Thank you for agreeing to participate in this study. The interview should take around an hour to complete, and we will do what we can to stay within this time limit and no longer. If you wish to discuss your experiences further after the interview is over, there will be a counselor on-site here at Elizabeth Fry you may speak with.

If you have difficulty understanding any of the questions, you can ask for clarification or request that I ask the question differently. If you wish to skip any questions, just say so.

[Start Audio Recorder] This is Emilie Macleod and it is [Date]. I am about to interview [Participant ID] at [Interview Location].

**Part 1: Demographic Information**

In this section of the interview I will ask you some basic demographic information.

1. How old are you?

2. What is your race or ethnicity?

3. How do you identify in terms of gender or sexuality? (note: you are not required to disclose cisgender or transgender status unless you want to)

4. What is your current housing situation?

5. What is the highest level of schooling you have completed?

6. Are you currently employed?

7. What is your approximate annual income?

8. Were you born in Canada? Are you a second-generation immigrant?

9. Do you have a health card?

10. Do you have any diagnosed mental illnesses, chronic illnesses, or disabilities? If you feel comfortable doing so, can you state what they are?

11. Do you take any non-opioid medication? If you are comfortable disclosing, what?

**Part 2: Experiences with Opioids**

Now I’m going to ask you about your experiences with opioids. While this information will be helpful in the context of understanding how this may impact whether or how you access naloxone, I will remind you that you are allowed to skip any questions you do not feel comfortable answering, and that you are allowed to pause the interview if you feel distressed or stop the interview if wish to withdraw.

1. Please tell me about your use of opioids.

Probes: What kind of opioids have you used? Do you use prescription or non-prescription opioids or both?How often do you use opioids? Are there times when you use more/less often? Can you describe why the frequency of use might change for you?

2. Have you ever taken opioids with other substances, such as alcohol or other prescription or non-prescriptions drugs?

Probes: If so, when, and with which substances?

3. Can you think of some reasons why you might mix opioids with other drugs? Can you tell me a bit about the reasons?

4. Can you describe the circumstances that led you to begin using opioids?

Probes: How old were you? Was someone with you? If you injected, who, if anyone, taught you how to use the equipment?

5. How do you feel about your opioid use?

6. Are other people in your life aware you take opioids? Can you tell me what it is like to know that these people know of your opioid use?

Probes: Did you share with them that you use opioids? Did they find out by accident? Please tell me about this?

7. Do you usually take opioids alone, or with other people? Can you tell me about that?

Probes: Why do you take opioids [alone, with other people]? If you take them with other people present, are they usually family? Friends? Strangers? Other?

8. This might be difficult for you to talk about, but I’d like to ask you about any experiences of overdose you may have had. Remember you can decline to answer or ask me to come back to the question later in the interview. Can you tell me if you have ever overdosed? If you have, can you please tell me about that experience(s)?

Probes: Was your overdose accidental or intentional?

9. Do you know anyone who has overdosed? If so, can you describe the experience(s)? [if multiple, ask about most difficult or most recent.]

Probes: Was it family member, friend, stranger, other? Did you know how to help the person?

**Part 3: Experiences with Naloxone**

This section of the interview will focus on experiences you have had related to naloxone, an opioid overdose antidote that is available in Ontario. The environment surrounding naloxone has shifted significantly in the past couple of years, and it is important to find out whether current methods are actually helping people who use opioids who have need of naloxone.

1. Can you tell me how you first learned about naloxone?

2. Do you have a naloxone kit? If so, where did you get it.

Probes: Who taught you how to use it? If you don’t have a kit right now, is there a reason? Do you feel safe when you attempt to access naloxone?

3. Have you ever gotten a naloxone kit from a pharmacy? If so, how was your experience? If not, why not?

4. Have you ever gotten a naloxone kit from a community health service or other type of service?

Probes: What type of service? What was it like to access naloxone from this service?

5. Has anyone ever used naloxone on you? If so, can you able to describe the experience for me?

6. Have you ever used naloxone on someone else? If so, are you able to describe the experience for me?

7. Have you ever needed naloxone, for yourself or for anyone else, but not had any?

8. What dosage forms of naloxone do you have experience with (syringe, nasal spray, autoinjector, etc.)? What do you find effective or ineffective about these forms?

9. In the event of an opioid-related emergency, would you feel safe asking for help?

Probes: For yourself? For others?

**Part 4: Legal and Social Perceptions**

We are nearing the end of the interview. I have a few more questions about naloxone.

1. Accessing services may be positive, negative, or neutral. I would like to ask you if, as a woman who uses opioids, that you feel that your gender/gender identity impacts the way you access services. If you are a trans woman you may choose to disclose this and contextualize your answer in terms of your experiences as a transgender person, in addition to being a woman, but it not required. Do you feel that your gender/gender identity impacts the way you access the following services and if so, how and why?

- Naloxone services
- Other harm reduction services
- Emergency department services
- Other health or opioid-related services (e.g. primary care physician, methadone clinics).

2. What is your view on legal attitudes surrounding opioids? The criminalization of certain opioids (e.g. heroin)?

3. How do you feel about law enforcement, fire services, paramedics carrying naloxone?

4. In general, how do you view the government’s response to the opioid crisis in Ontario/Canada? Comparatively, how do you view Toronto’s response?

5. Do you think opioid overdoses in Ontario should be considered a provincial emergency?
